# Enhanced Monitoring of Urethral and Bladder Mobility in Postpartum Stress Urinary Incontinence using Combined Ultrasound Techniques

**DOI:** 10.2174/0115734056319919250604093316

**Published:** 2025-06-13

**Authors:** Hai-Ying Gong, Hong-Yun Zhang, Ting-Ting Cui, Jiang Zhu

**Affiliations:** 1 Department of Diagnostic Ultrasound, Women’s Hospital, School of Medicine, Zhejiang University, Zhejiang Provincial Clinical Research Center for Obstetrics and Gynecology, Hangzhou, China; 2 Department of Diagnostic Ultrasound, Yiwu Traditional Chinese Medicine Hospital, Zhejiang, China; 3 Department of Diagnostic Ultrasound, Taizhou Traditional Chinese Medicine Hospital, Zhejiang, China

**Keywords:** Biplanar ultrasound, Pelvic floor ultrasound, Postpartum, Stress urinary incontinence, Transperineal ultrasound

## Abstract

**Objective:**

This study aimed to compare the consistency between smart pelvic floor ultrasound and biplanar transrectal ultrasound in detecting early stress urinary incontinence (SUI) by assessing urethral dilation and bladder structure.

**Methods:**

We selected 40 multiparas who went through prenatal assessment after delivery and had standard pelvic floor ultrasounds at 6 weeks after childbirth, spanning from June 2022 to September 2022. The Bland-Altman method was employed to evaluate the consistency between biplanar transrectal ultrasound and transperineal pelvic floor ultrasound in assessing the mobility of the bladder neck and the posterior bladder wall in women.

**Results:**

Biplanar transrectal ultrasound and transperineal pelvic floor ultrasound demonstrated strong consistency in evaluating bladder neck and posterior bladder wall mobility in women (P>0.05). The analysis of each pelvic floor observation index using Bland-Altman plots indicated that approximately 97.5% of data points fell within the 95% consistency limit.

**Conclusion:**

Our findings suggest that biplanar transrectal ultrasound is a reliable supplementary method to transperineal pelvic floor ultrasound for diagnosing SUI.

## 1 INTRODUCTION

Stress urinary incontinence (SUI) is one of the most prevalent pelvic floor disorders affecting women’s health, with an incidence rate ranging from 15.1% to 59.7% [1, 2]. While not life-threatening, SUI significantly impacts social activities and the overall physical and mental well-being of women. Early and well-timed pelvic floor rehabilitation exercises have shown efficacy in improving pelvic floor muscle function and preventing SUI [3]. The postpartum period (*i.e*., 6 to 12 weeks following childbirth) is critical for effective rehabilitation. Invasive urodynamic examination, typically reserved for complex preoperative cases, is regarded as the gold standard despite limited high-level evidence supporting its use [4]. Simpler methods, such as questionnaires and non-invasive evaluations, are preferred in clinical practice, although they are time-consuming. The condition of urinary leakage can be assessed using a cystoscope during coughing [5]. If the bladder neck is fully closed, SUI will not occur; however, if the bladder neck remains partially open, SUI is likely to be present.

Transperineal ultrasound (TPUS) is a novel, non-invasive approach endorsed by the International Urogynecological Association and the American Institute of Ultrasound in Medicine for SUI evaluation [6]. TPUS provides real-time visualization of bladder neck and proximal urethra changes under increasing intra-abdominal pressure. However, discrepancies between ultrasound findings and symptom severity warrant further investigation [7]. Conventional three-dimensional pelvic floor ultrasound often fails to detect urethral dilatation in cases of urinary incontinence with visible leakage. To address this limitation, we explored a combination of transperineal three-dimensional ultrasound and biplanar transrectal ultrasound. This approach aimed to identify individuals at high risk for SUI, facilitating early diagnosis and intervention. Additionally, we assessed the clinical significance of this approach in diagnosing SUI, paving the way for innovative SUI screening and diagnosis techniques.

## 2 MATERIALS AND METHODS

### 2.1 Participants

In this cross-sectional study, we randomly collected clinical information of 40 female patients who were receiving medical care at the outpatient clinics of the Women’s Hospital, School of Medicine, Zhejiang University Hospital affiliated with Zhejiang University School of Medicine using a simple randomization method. The effectiveness of the randomization was validated based on clinical symptoms and imaging characteristics. The data collection period spanned from June 2022 to September 2022. After conducting clinical assessments and utilizing medical imaging, we identified 28 female patients with SUI, among whom 8 exhibited a formation of an internal urethral orifice funnel (accounting for 28.6% of this subgroup). Additionally, 12 cases were without SUI. This study was conducted in accordance with the Declaration of Helsinki and approved by the Ethics Committee of Women’s Hospital, School of Medicine, Zhejiang University (IRB-20200087-R). A written informed consent was obtained from all participants.

To analyze the ultrasound characteristics of bladder neck and posterior bladder wall mobility in women with and without SUI, we employed both transperineal pelvic floor ultrasound and biplanar transrectal ultrasound. These imaging techniques were used to observe the changes in these areas during resting and Valsalva states, with a particular focus on the internal urethral orifice funnel.

#### 2.1.1 Inclusion Criteria

Female participants who met the following criteria were eligible for inclusion in this study:

1. Underwent either cesarean section or natural vaginal delivery.

2. Within the childbearing age range of 20 to 41 years.

3. No previous instances of SUI or pelvic surgical interventions apart from cesarean section.

4. No medical history of conditions like hypertension, diabetes, tuberculosis, and heart disease.

5. Revisited the Hospital of Zhejiang University School of Medicine for a postpartum follow-up at the 6-week mark.

6. Capable of comprehending and accurately responding to the questionnaire.

7. Expressed willingness to actively take part in this study.

#### 2.1.2 Exclusion Criteria

Female participants who met any of the following criteria were excluded:

1. Aged below 20 years or above 41 years.

2. Comorbidities like ascites or other ailments leading to increased abdominal pressure, along with urinary tract infections and tumors of the urinary system.

3. Prior respiratory issues like chronic cough and asthma.

4. Unfavorable lifestyle habits, such as smoking and alcoholism.

5. Could not recall questionnaire information accurately.

6. History of combined urinary and/or reproductive disorders.

7. Experiencing overflow or neurogenic incontinence.

8. Incapable of effectively performing a maximal Valsalva maneuver.

9. Inadequate bowel preparation that hindered the visibility of urethral structures.

10. Intestinal conditions like hemorrhoids preventing the use of transrectal ultrasound.

11 .Women who declined participation in the study.

### 2.2 Instruments

In this study, the imaging device utilized for color Doppler ultrasound was the Mindray NueWa R9. The scanning tools included the D8-2U probe with three-dimensional volumetric capabilities (operating at a frequency range of 2.0 to 7.3 MHz), along with the ELC13-14U biplanar transrectal probe designed for high-frequency ultrasound imaging. A quiet, interference-free environment was selected for calibration, with stable temperature and moderate relative humidity ensured. Calibration was avoided in harsh conditions, including strong electromagnetic fields, high temperatures, and high humidity. During the calibration process, good coupling was maintained between the probe and the calibration block to prevent air gaps that could influence measurement results. Ultrasound equipment was regularly maintained to ensure stability and accuracy, and professional calibration was performed annually by a certified company. The frequency of these procedures is adjusted based on the patient's condition.

### 2.3 Study Methods

#### 2.3.1 Data Collection

Data was collected using a pre-formulated questionnaire containing questions on age, body mass index, past childbirths, delivery method, newborn birth weights, and the existence of bladder prolapse and SUI (**supplementary material**). The questionnaire survey was completed without collecting any personal information, and participants consented to provide accurate information. The data collection process involved reviewing the completeness and accuracy of the responses to ensure participants' comprehension. After being validated, the questionnaires were retrieved. Incomplete or inaccurate questionnaires were marked and categorized according to the severity of issues, such as “minorly incomplete” (missing one or two items), “severely incomplete” (missing over two items), or “inaccurate” (containing obvious errors or contradictory answers). For questionnaires marked as minorly incomplete, an attempt was made to contact respondents *via* email, phone, or text message to request the missing information. If respondents could not be reached and the information was minorly incomplete, data from other similar respondents were considered as substitutes (ensuring the representativeness and accuracy of the substitute data). For severely incomplete questionnaires, exclusion was generally chosen, as these questionnaires likely lacked sufficient information to support the research conclusions. Those containing obvious errors or contradictory answers were also excluded to prevent misleading analysis results.

Missing data were handled using the following strategies: (1) The reasons for missing data (*e.g.*, technical failures, participant refusal) were meticulously documented. (2) For randomly missing data, multiple imputation techniques were applied. These techniques generated several plausible imputed values based on existing data, and the results from analyzing these imputed datasets were combined to produce more robust conclusions. (3) The impact of missing data on study outcomes was assessed by comparing analyses of the complete dataset with those of the imputed dataset, exploring potential biases introduced by missing data.

#### 2.3.2 “Gold Standard” Approach to the Examination of Research Data

We observed the mobility of the bladder neck and posterior bladder wall during rest and the Valsalva maneuver in participants using the following probes: the transperineal pelvic floor volume probe and the biplanar transrectal probe. Specifically, by measuring the lowest point of the posterior wall of the bladder, a horizontal line passing through the posterior and inferior edge of the symphysis pubis (SP) was used as the reference point. Participants were instructed to perform the Valsalva maneuver, and measurements were taken: (1) If the measured point is more than 1 cm above the reference line, there is no bladder prolapse. (2) If the measured point is within 1 cm above the reference line, it is confirmed as Grade I bladder prolapse. (3) If the measured point is within 2 cm, it is confirmed as Grade II bladder prolapse.

##### 2.3.2.1 Bladder Neck Mobility

 The mobility of the bladder neck was measured using transrectal and transperineal ultrasound images to observe positional changes of the bladder neck in different states (*e.g.*, at rest and during the Valsalva maneuver). The mobility is typically expressed in millimeters (mm). The degree of movement was calculated by comparing the bladder neck's position at rest and during the Valsalva maneuver [8].

##### 2.3.2.2 Posterior Bladder Wall Mobility

The mobility of the posterior bladder wall was assessed by measuring its movement during rest and the Valsalva maneuver. This parameter helps evaluate the compliance of the bladder wall and the overall function of the bladder.

The measurement obtained from the transperineal pelvic floor volume probe was considered the reference standard (“gold standard”), while the measurement from the biplanar transrectal probe served as an observational measurement. Our focus was to compare the consistency of these two methods for pelvic floor examination in women six weeks after giving birth [8].

#### 2.3.3 Bias Control

Researcher bias control was implemented through the following measures: (1) rigorously adhere to the inclusion and exclusion criteria when selecting or excluding relevant participants; (2) meticulously validate the questionnaire for completeness and accuracy of information; (3) ensure meticulous data entry; and (4) maintain a strong commitment to keeping participant information strictly confidential.

Study participant bias control was implemented through the following measures: (1) ensure comprehension of questionnaire content and encourage truthful and effective responses, and (2) promote clear and honest recall of the questions to be answered.

To ensure the study design was rigorous and the sample selection was representative, clear inclusion and exclusion criteria were established to accurately reflect the characteristics of the target population while minimizing selection bias. Delivery information was meticulously recorded and verified, using participants' recollections supplemented by medical records or related documentation to confirm delivery details and methods. For those unable to provide medical records, alternative verification methods, such as hospital archive searches or family member confirmation, were employed. Standardized and validated questionnaires were used to collect reliable self-reported data on delivery, and professional training was provided to interviewers to ensure effective techniques for guiding participants in accurately recalling and reporting relevant details. Furthermore, objective assessment tools, including intelligent pelvic floor ultrasound and biplanar transrectal ultrasound, were utilized to monitor urethral opening and bladder activity in real-time. These objective data were cross-validated with self-reported questionnaire responses to identify and correct potential recall bias, enhancing the overall reliability and accuracy of the study findings.

By utilizing a combination of methods, such as standardized questionnaires, objective test validation, clinical assessments, follow-up and repeat testing, training and education, and statistical analysis techniques, the accuracy of self-reported data can be significantly improved. These measures not only help ensure the reliability of research findings but also provide robust data support for the monitoring and treatment of postpartum stress urinary incontinence.

#### 2.3.4 Diagnostic Criteria for SUI and Cystocele

The criteria used to diagnose SUI are outlined as follows: SUI is defined by the medical community as the unintentional release of urine through the urethra without a sensation of urgent need to urinate, caused by situations that elevate abdominal pressure, like coughing, sneezing, or vigorous physical activity. When examining the bladder during filling, urodynamic testing shows only an increase in abdominal pressure without contractions of the detrusor muscle in patients with SUI. In this study, the required conditions for confirming an SUI diagnosis were: (1) involuntary urine leakage due to a sudden rise in abdominal pressure from actions like coughing, laughing, sneezing, or intense exercise; (2) a positive outcome in either the Marshall-Marchetti test and/or swab test; (3) urodynamic testing results that corroborated the presence of SUI. According to the Ingelman-Sundberg scale, SUI severity is classified as mild, moderate, or severe [9-11]: (1) mild incontinence occurs during sneezing and coughing, necessitating no more than minimal protective measures; (2) moderate incontinence arises during routine activities, such as brisk walking, running, or jumping, requiring some form of protective measures; (3) severe incontinence happens even during light activities and changes in body position while lying down. The diagnostic criteria for cystocele were defined as follows: the measurement of cystocele took place at the lowest spot of the posterior bladder wall, using the horizontal line that goes through the back and lower edge of the symphysis pubis (SP) as a point of reference. Specifically, the study participants were instructed to perform Valsalva maneuvers, and during the maximum exertion of the Valsalva maneuver: (1) there was no cystocele if the measurement point was situated more than 1 cm above the reference line; (2) a grade I cystocele was confirmed if the measurement point was within 1 cm above the reference line; (3) a grade II cystocele was confirmed if the measurement point was within 2 cm below the reference line; and (4) a grade III cystocele was confirmed if the measurement point was more than 2 cm below the reference line [12].

#### 2.3.5 Pelvic Floor Ultrasound

The ultrasound measurements were conducted using a double-blind method. The participants in the study were directed to empty their bladder and wait for a period of 10 to 15 minutes, during which the amount of urine remaining in the bladder was typically under 50 mL.

During the Valsalva maneuver, the participants took a deep breath and then held it while exerting downward pressure on the abdomen. This maneuver was sustained for at least 6 seconds.

**Fig. (1):**
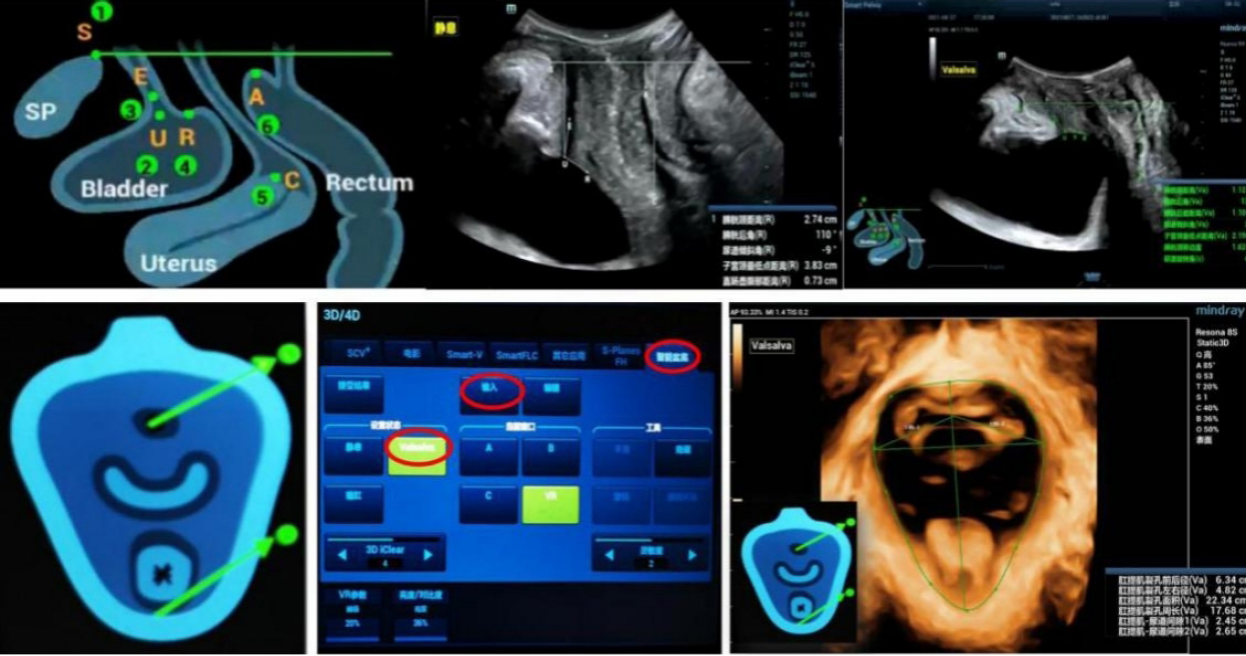
Schematic illustration of ultrasound measurement of pelvic floor function. Adobe illustrator images of the midsagittal plane obtained by transperineal pelvic floor ultrasound at rest and Valsalva states. (SP: symphysis pubis; UR: internal urethral orifice; E: bladder neck; C: neck of the uterus; A: rectum and anal canal)

The examination procedure was as follows: The specific settings for pelvic floor ultrasound were chosen, and the participants were positioned in the lithotomy position. The surface of the ultrasound probe was coated with sterile neutral gel. After separating the labium majora on both sides, the upper part of the probe was positioned near the lower edge of the SP and placed against the perineum. The participants were advised to breathe calmly, and the probe was gently placed at the vaginal opening, avoiding excessive pressure, to obtain a mid-sagittal view of the pelvic floor. This view clearly displayed structures, such as the SP, bladder, urethra, vagina, and anal canal from front to back. The “3D” button was pressed to generate a three-dimensional volume image of the pelvic floor in its resting state, and this image was saved in the device. The “automated pelvic floor” feature was activated, and the labeled points were marked based on the provided schematic diagram: S represented the posterior lower edge of the SP, P indicated the distal middle axis of the SP, U denoted the junction of the urethra and bladder, E marked the proximal middle axis of the urethra, and R symbolized the posterior bladder wall near the urethra (Fig. **1**). Images of the SP, bladder, and urethra were captured in the mid-sagittal plane during both resting and Valsalva states for measurement. The spatial relationship between the lowest point of the posterior bladder wall and the horizontal line of the posterior lower edge of the SP was measured, and the presence or absence of a cystocele was noted when the participants performed a maximal Valsalva maneuver. Ultrasound images showing the mobility of the bladder neck and posterior bladder wall were recorded in both resting and Valsalva states, and the openness of the urethra was also observed.

Afterward, a biplanar transrectal ultrasound was conducted. Study participants were positioned in the lithotomy position, and the probe, wrapped in a double-layer condom, was gradually introduced into the rectum.

(1) At resting state: Initially, the high-frequency linear array mode was employed. In this mode, the probe emitted a rectangular acoustic beam laterally. This setting allowed visualization of the sagittal cross-section of the middle and lower segments of the vaginal area, positioned between the anterior wall of the rectum and the posterior wall of the urethra. This technique was utilized to distinctly exhibit the anterior wall of the rectum, posterior wall of the vagina, anterior wall of the vagina, posterior wall of the urethra, urethra, anterior wall of the urethra, and the SP in a progression from the far field to the near field (Fig. **2A**). Subsequently, the measurement of the distance from the bladder neck to the lower edge of the SP was carried out, along with the observation of the internal opening of the urethra. The measurement of the distance between the urethra and the lower border of the SP was also conducted.

(2) At the Valsalva state: while the study participants were engaged in a maximal Valsalva maneuver, the distance between the bladder neck and the lower edge of the SP was gauged. The presence or absence of the internal urethral orifice opening was observed (Fig. **2B**), along with measurement of its length and width. Additionally, the absence of cystocele and its specific type were noted.

All assessments were conducted by a highly experienced senior obstetrician/gynecology sonographer with a minimum of 5 years of experience. This specialist obtained standard cross-sectional images and stored them within the ultrasound device according to the criteria outlined above.

To ensure the accuracy and consistency of the measurements, the following standardized procedures were established: (1) All participants were required to empty their bladder before the examination to minimize the influence of residual urine on the results. (2) Participants were placed in the lithotomy position to ensure consistency in posture during the examination. (3) The intelligent pelvic floor ultrasound probe and the biplane transrectal ultrasound probe were positioned in their designated locations, ensuring proper contact with tissues to minimize measurement errors. (4) During the examination, participants were instructed to perform a series of actions, such as coughing and breath-holding, to observe the dynamic changes in urethral opening and bladder mobility. In addition, before the examination, observational indicators for urethral opening and bladder mobility, such as bladder neck mobility and urethral rotation angles, were clearly defined. Uniform measurement standards and methods were developed to ensure comparability of results across different observers. Lastly, all observers underwent standardized training to familiarize themselves with the examination process and observational indicators, reducing inter-observer variability.

The ultrasound examinations, including transperineal and transrectal ultrasound, were performed by an experienced gynecologist to ensure consistency and continuity in the process. If multiple physicians conducted the examinations, a standardized evaluation protocol was followed to maintain result consistency. In cases of diagnostic discrepancies, consensus was achieved through group discussions, re-evaluations, or by involving a third-party expert for review.

### 2.4 Statistical Methods

The sample size was determined as follows:

The significance level (α) was set at 0.05, and the statistical power of the test (β) was chosen at 0.80. It is assumed that the positive rate for biplanar transrectal ultrasound is p1 = 0.7, while the positive rate for transperineal pelvic floor ultrasound is p2 = 0.4.

In the above formula, Z_α/2_ is the critical value from the normal distribution table corresponding to the significance level (when α = 0.05, the Z value is approximately 1.96), and Z_β_ is the Z value corresponding to the statistical power (when β = 0.20, the Z value is approximately 0.84). Based on this formula, the total sample size needed was approximately n ≈ 39.5. Therefore, 40 cases were selected for each group.

Data analysis was conducted using SPSS 21.0 and Medcalc 20.1.14 software. Measurement data are expressed as mean ± standard deviation (X ± S) and compared using the t-test. Comparisons of rates among count data were carried out using the chi-squared test. The consistency of the outcomes of the two measurement techniques was compared by Bland-Altman analysis using Medcalc 20.1.14. A difference of P < 0.05 was statistically significant.

## 3 RESULTS

The 6^th^ edition of the International Continence Society book (2017) points out that many SUI patients exhibit urethral activity, although it remains unclear what aspect of this activity leads to urethral opening during stress [13]. Transrectal ultrasound plays an important role in detecting early and long-term urinary incontinence and serves as an independent prognostic factor. The contractile function of the external urethral sphincter is significantly associated with urinary incontinence [13,14]. This study involved 40 study participants (age range: 23 - 36 years; median age, 29 years). Both transperineal pelvic floor ultrasound and biplanar transrectal ultrasound were conducted on all 40 participants to assess the pelvic floor.

### 3.1 Statistical Results of the Paired T-Test for the Two Measurement Methods

#### 3.1.1 Comparisons of Bladder Neck Distances and Posterior Bladder Wall Distances at the Resting State

At rest, the distance of the bladder neck from the lower margin of the SP was 2.55 ± 0.46 cm as measured by transperineal pelvic floor ultrasound and 2.45 ± 0.76 cm as measured by biplanar transrectal ultrasound. There was no statistically significant difference in the measurement values (t = 0.920, P = 0.363). The distance of the posterior wall of the bladder, as determined by transperineal pelvic floor ultrasound, was recorded as 2.60 ± 0.40 cm. Similarly, using biplanar transrectal ultrasound, this distance was measured at 2.45 ± 0.72 cm. No statistically significant difference was observed (t = 1.333, P = 0.190) (Table **1**).

#### 3.1.2 Comparisons of Bladder Neck Distances and Posterior Bladder Wall Distances at the Valsalva State

At the Valsalva state, the distance of the bladder neck was recorded as 0.51 ± 0.19 cm using transperineal pelvic floor ultrasound, while biplanar transrectal ultrasound yielded a measurement of 0.43 ± 0.22 cm. There was no statistically significant difference in the measurement values (t = 0.704, P = 0.485) (Table **1**). The posterior bladder wall distance measured by transperineal pelvic floor ultrasound was 0.43 ± 0.23 cm, and the distance measured by biplanar transrectal ultrasound was 0.34 ± 0.26 cm. No significant difference was observed (t = 0.657, P = 0.515) (Table **1**).

### 3.2 Bland-Altman Analysis of Assessing the Consistency between Two Measurement Methods

#### 3.2.1 Bladder Neck Distance at the Resting State of the Study Participants

**Fig. (3):**
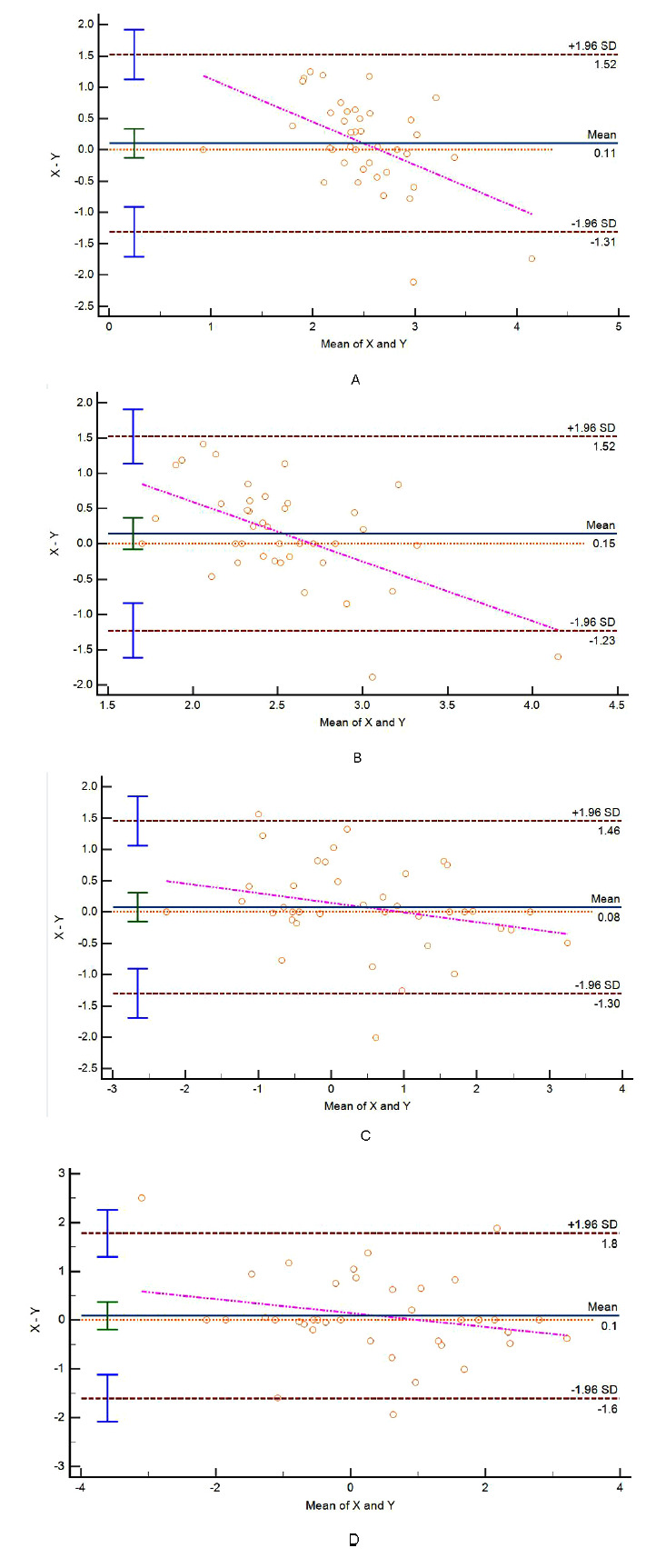
**A**) Results from Bland-Altman analysis of consistency in terms of bladder neck distances at the resting state. **B**) Results from Bland-Altman analysis of consistency in terms of posterior bladder wall distances at the resting state. **C**) Results from Bland-Altman analysis of consistency in terms of bladder neck distances at the Valsalva state. **D**) Results from Bland-Altman analysis of consistency in terms of posterior bladder wall distances at the Valsalva state.

At rest, the mean value of the difference between the two measurement values of the bladder neck distance from the inferior margin of the SP was 0.1050 ± 0.114 cm, with 95% limits of agreement (LoA) for the difference of -1.3102 and 1.5202 and a confidence interval (CI) for the 95% LoA of -1.7082 and 1.9182. The Bland-Altman analysis demonstrated that only 1/40 of the data were outside the CI of the 95% LoA. Accordingly, the two measurement methods have good consistency in examination (Fig. **3A**).

#### 3.2.2 Posterior Bladder Wall Distances at Rest in 40 Study Participants

At rest, the mean difference between the two measurement values of the posterior bladder wall distance was 0.148 ± 0.111 cm. The 95% LoA of the difference was -1.2284 and 1.5244, and the CI of the 95% LoA was -1.6154 and 1.9114. According to Bland-Altman analysis results, only 1/40 of the data were located outside the CI of the 95% LoA. In summary, the examination consistency between the two measurement methods was relatively high (Fig. **3B**).

#### 3.2.3 Bladder Neck Distance at the Valsalva State in 40 Study Participants

At the Valsalva state, the mean of the difference between the measurement values of bladder neck distance was 0.079 ± 0.112 cm. The 95% LoA of the difference was -1.2975 and 1.4559, and the CI of the 95% LoA was -1.6899 and 1.8484. It was observed through Bland-Altman analysis that only 1/40 of the data were outside the CI of the 95% LoA. Hence, a positive agreement was found in examination consistency between the two measurement techniques (Fig. **3C**).

#### 3.2.4 Posterior Bladder Wall Distance at the Valsalva State in 40 Study Participants

At the Valsalva state, the difference between the measurement values of posterior bladder wall distance had a mean value of 0.091 ± 0.138 cm, with the 95% LoA of -1.6007 and 1.7823 and a CI of 95% LoA of -2.0829 and 2.2645. The Bland-Altman analysis exhibited only 1/40 of the data outside the CI of the 95% LoA. In conclusion, the two measurement techniques demonstrated strong consistency in their results (Fig. **3D**).

The above findings suggest that symptoms of female lower urinary tract disorders are often accompanied by abnormalities in the urethra and/or pelvic cavity during urination [15]. Transrectal ultrasound can clearly detect these abnormalities and can be used for the objective evaluation of female lower urinary tract symptoms. The results highlight the advantages of both transrectal and transperineal examinations.

#### 3.2.5 Ultrasound Image Conditions of the 40 Study Participants at Resting and Valsalva States

Out of the 40 study participants, 28 experienced SUI, while 12 did not encounter SUI. During periods of rest and the Valsalva maneuver, the pelvic floor tissues were distinctly observable in both participants with and without SUI, as depicted in Figs. (**4** and **5**). Moreover, participants in both groups were capable of successfully performing the Valsalva maneuver with separate guidance. Furthermore, evident alterations in the structure of pelvic floor tissues were observable in participants with and without SUI both before and after the execution of the Valsalva maneuver. Following the Valsalva maneuver, the bladder neck, posterior bladder wall, proximal urethra, and surrounding supportive tissues all exhibited posterior and inferior displacement in both groups.

## 4 DISCUSSION

### 4.1 Main Findings

This study explored the relationship between spatial movements of the bladder neck and proximal urethra, as well as changes in the posterior vesical angle. The findings contribute to the diagnosis of SUI and introduce a novel diagnostic approach.

### 4.2 Transperineal Ultrasound

Transperineal ultrasound is an economical technique for assessing pelvic prolapse, widely used in clinical practice due to its safety, affordability, and absence of ionizing radiation [16]. This method enables real-time evaluation of the pelvic floor dynamics. Nonetheless, the standard three-dimensional pelvic floor ultrasound has limitations, as it cannot effectively measure urethral dilatation in certain incontinence cases characterized by substantial urine leakage [7]. One such solution is the implementation of a biplanar transrectal/transvaginal probe. This probe permits the emitted beam to be directed perpendicularly to the vaginal wall for imaging purposes [17]. Additionally, the biplanar transrectal probe operates at a relatively high frequency of 13 to 14 MHz, resulting in significantly superior resolution compared to the transperineal probe, enabling clearer visualization of bladder and urethra structures. Biplanar transperineal ultrasound allows for multi-directional detection of the pelvic floor, offering a new approach to pelvic floor examination beyond traditional methods.

### 4.3 Biplanar Transrectal Probe

Three-dimensional transperineal ultrasound and biplanar transrectal probe represent novel screening methods. Bladder neck mobility has been identified as a potential diagnostic marker for early SUI in women [18]. We assessed the distance between the bladder neck and the lower edge of the SP utilizing transperineal pelvic floor ultrasound and biplanar transrectal ultrasound. The CIs of 95% LoA for resting and Valsalva states were -1.7082 and 1.9182 and -1.6899 and 1.8484, respectively, for the two methods. These findings highlight strong consistency in examination outcomes between the two measurement approaches, suggesting that the mentioned parameters can serve as effective diagnostic indicators for SUI in women. A recent study confirmed that relying solely on bladder neck descent is insufficient for a comprehensive evaluation of SUI. Consistent with our findings, a Chinese report indicated that the specificity of BND in diagnosing SUI is only 68.9% [19, 20]. Therefore, it is essential to explore abnormalities in urethral support structures to better understand the etiology and pathogenesis of SUI. Most studies have focused on bladder neck mobility, with limited information provided about the rest of the urethra [21]. Our study demonstrated that biplane transrectal ultrasound can clearly visualize the bladder wall, the internal urethral orifice, and surrounding tissues.

As traditional three-dimensional ultrasound imaging of the pelvic floor cannot effectively evaluate urethral dilatation in certain incontinent individuals, even with substantial urine leakage, the biplanar transrectal probe offers a distinct ability to clearly observe the urethral funnel from start to end. The underlying mechanisms of SUI involve various factors, such as the downward movement of the bladder neck and proximal urethra, impaired closure function of the urethral lining, and reduced activity of the intrinsic urethral sphincter [22]. Our study focused on investigating the distances between the bladder neck and the posterior bladder wall, both with and without the opening of the urethra in individuals with SUI, using transrectal and pelvic floor ultrasound. We found a strong agreement between the two measurement methods in terms of assessing the bladder neck and posterior bladder wall distances during rest and the Valsalva maneuver.

The bladder neck descent method was used to assess the mobility and position of the bladder neck, which was consistent with most previous studies. Our study demonstrated that a noticeable increase in bladder neck mobility was linked to transvaginal childbirth. This heightened mobility of the bladder neck, along with reduced pressure for closing the urethra, is associated with SUI and is exacerbated after vaginal delivery [23].

During urination, the pressure within the bladder is higher than in the urethra. When the pressure in the abdomen rises, it causes the anterior and posterior vaginal walls to be pushed together. Simultaneously, the proximal urethra and bladder neck experience pressure from the abdomen, while the levator ani muscle contracts to close the external urethral opening and the urethra. This mechanism ensures control over urinary release as abdominal pressure increases [24]. However, if the angle between the posterior urethra and bladder increases, or the angle of urethral rotation becomes larger during the Valsalva maneuver, the heightened abdominal pressure is not equally transmitted to both the bladder neck and the proximal urethra. Instead, the pressure is mainly applied to the bladder, resulting in higher pressure within the bladder compared to the urethra. This imbalance leads to SUI [25-27].

Contemporary techniques for assessing structures within the pelvic floor encompass the utilization of magnetic resonance imaging (MRI) and ultrasound imaging. An investigation into these imaging methods highlighted notable distinctions between ultrasound and MRI in assessing both the descent of organs and gap dilation [28]. Employing MRI for pelvic floor examination enables a visual assessment of modifications in microstructures, such as the urethral sphincter muscles and levator ani muscles, from various angles [29]. The primary underlying causes of SUI involve an increase in the mobility of the urethra and dysfunction of the urethral sphincter [22]. The term “urethral funnel” describes the distinctive widening of the internal opening of the urethra when the Valsalva maneuver is performed, indicating a significant indicator of impaired urethral sphincter function [30, 31]. Women experiencing SUI following childbirth tend to exhibit a lower position of the bladder neck and increased urethral mobility [32]. Our study showed that using pelvic floor ultrasound in conjunction with biplanar transrectal ultrasound to assess the urethra could serve as a supplementary diagnostic approach for SUI, with strong agreement between the two techniques. Furthermore, we identified urethral funnel formation in 28.6% (8/28) of participants with SUI, which is in line with the results of a previous study [33].

The presence of a urethral funnel increases the risk of SUI, as it characterizes an acoustic image representing urethral sphincter dysfunction in individuals with this condition. Our findings revealed that using pelvic floor ultrasound and biplanar transrectal ultrasound, clear images of the internal urethral orifice and urethra could be obtained, regardless of whether individuals have SUI or not. We were able to observe the shape of the urethra and bladder both when the individuals were at rest and during the Valsalva maneuver. The agreement between these two measurement techniques was highly consistent.

### 4.4 Limitations

This study has some limitations. Due to time constraints, we may not have been able to collect sufficient data or conduct more in-depth analyses, which could affect the comprehensiveness of the findings. Additionally, the inherent limitations of the chosen methods may have resulted in outcomes that are either inaccurate or challenging to interpret. To further enhance the accuracy and reliability of the research, future studies could consider expanding the sample size, extending follow-up periods, and optimizing examination techniques to overcome these limitations. Additionally, conducting in-depth analyses of specific subgroups could provide a more precise evaluation of the value of intelligent pelvic floor ultrasound and biplane transrectal ultrasound in monitoring urethral opening and bladder activity in women with postpartum stress urinary incontinence. In future research, the validity of randomization can be further verified by reporting baseline characteristics for each group (*e.g.*, age, BMI, disease severity), including a larger sample size, and minimizing selection and measurement bias. This will help enhance the reliability and reproducibility of the study results.

## CONCLUSION

In conclusion, the use of multimodal ultrasound examinations (*e.g.*, ultrasound elastography, ultrasound microvascular flow) combined with advancements in molecular biology and genetics could provide deeper insights into the physiological mechanisms underlying postpartum SUI, offering a solid theoretical foundation for treatment. In the present study, dual-plane transrectal ultrasound and transperineal pelvic floor ultrasound demonstrated high consistency in evaluating female bladder neck and posterior bladder wall mobility, improving the credibility of clinical assessments and providing more stable diagnostic support for clinicians. Bland-Altman plot analysis showed a high concentration of data points between the two methods, indicating strong concordance and further validating their clinical applicability. The results of this study confirmed the reliability of intelligent pelvic floor ultrasound combined with dual-plane transrectal ultrasound in assessing pelvic floor function in postpartum SUI women, establishing a new benchmark for the application of ultrasound technology in monitoring pelvic floor disorders. Highly consistent and reliable evaluation methods enable clinicians to more accurately assess patients' pelvic floor functional status, facilitating the development of more personalized treatment plans for postpartum SUI patients and improving the precision of clinical decision-making.

## AUTHORS' CONTRIBUTIONS

H.Y.G.: and J.Z.: Conception and design of the research; J.Z.: and H.Y.Z.: Acquisition of data; H.Y.G.: and T.T.C.: Analysis and interpretation of the data; T.T.C.: Statistical analysis; H.Y.G.: and J.Z.: Writing of the manuscript; J.Z.: and H.Y.G.: Critical revision of the manuscript for intellectual content.

## Figures and Tables

**Fig. (2A):**
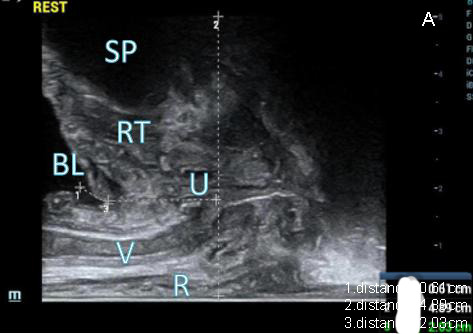
Midsagittal plane obtained by biplanar transrectal ultrasound at rest: normal pelvic floor image.

**Fig. (2B):**
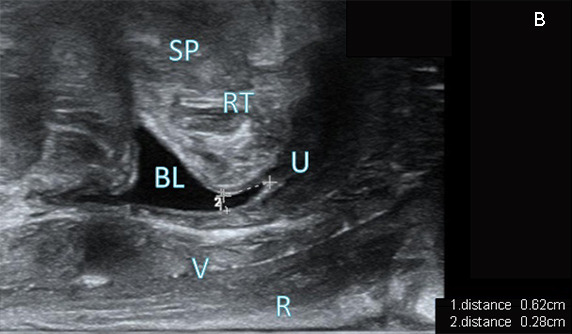
Midsagittal plane obtained by biplanar transrectal ultrasound at the Valsalva state: internal urethral orifice opening in individuals with SUI. (SP: symphysis pubis; RT: retropubic space; U: urethra; BL: bladder; V: vagina; R: anterior rectal wall)

**Fig. (4):**
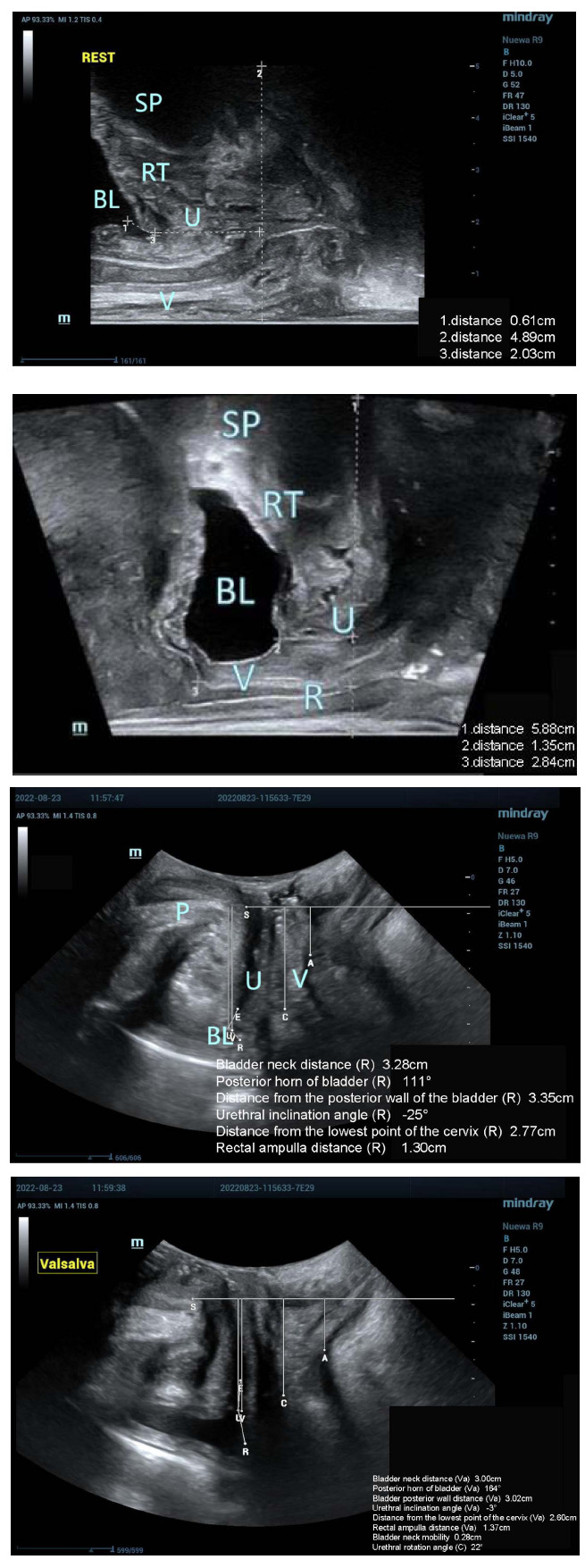
Ultrasound images of the study participants without SUI using the two ultrasound methods. Biplanar transrectal probe: (**A**) Resting state, no SUI; (**B**) Valsalva state, no SUI; Transperineal pelvic floor probe: (**C**) Resting state, no SUI; (**D**) Valsalva state, no SUI (SP: symphysis pubis; RT: retropubic space; U: urethra; BL: bladder; V: vagina)</unknown

**Fig. (5):**
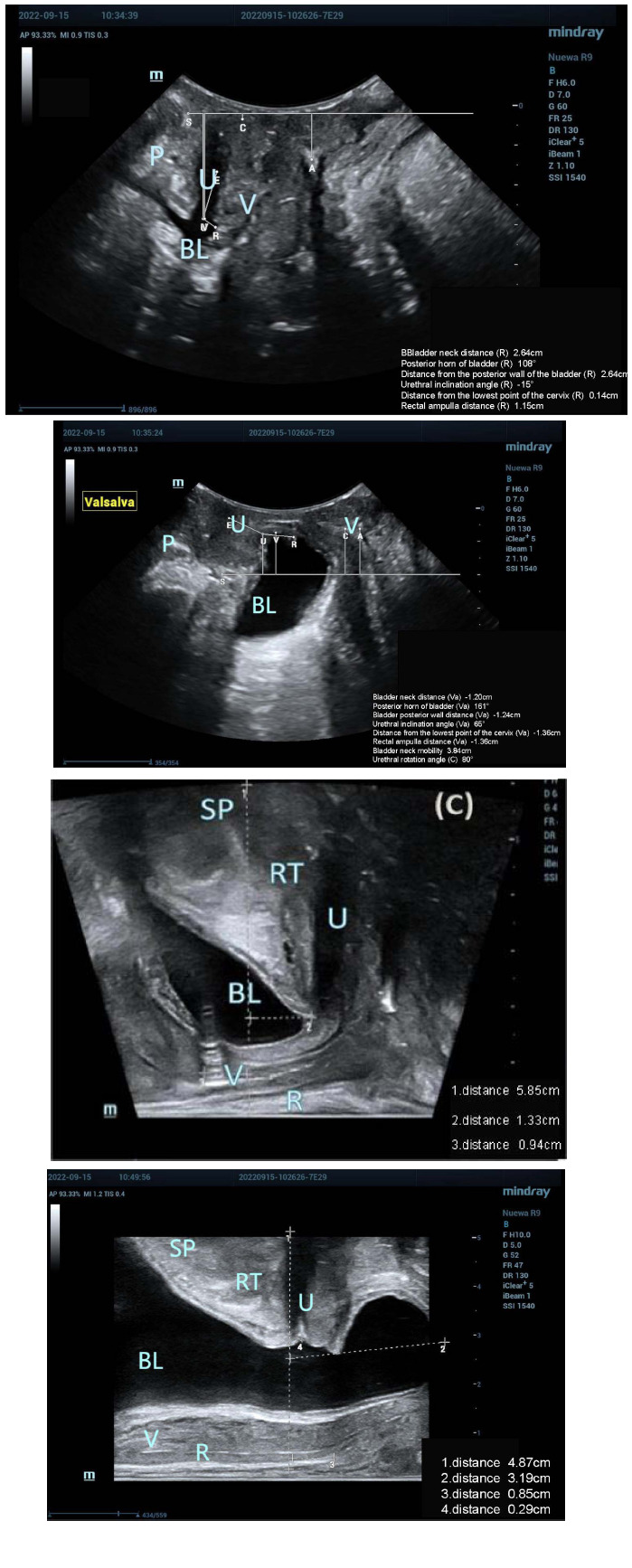
Ultrasound images of the study participants with SUI using the two ultrasound methods. Transperineal pelvic floor probe: (**A**) Resting state, SUI; (**B**) Valsalva state, SUI; Biplanar transrectal probe: (**C**) Resting state, SUI; (**D**) Valsalva state, SUI with cystocele (type III cystocele). (SP: symphysis pubis; RT: retropubic space; U: urethra; BL: bladder; V: vagina)

**Table 1:** Results of paired *t*-tests for ultrasound parameters of the participants examined using the two measurement methods.

-	At the Resting State		At the Valsalva State	
-	Bladder Neck Distances	Posterior Bladder Wall Distances	Bladder Neck Distances	Posterior Bladder Wall Distances
Transperineal pelvic floor ultrasound	2.55±0.46	2.60±0.40	0.51±0.19	0.43±0.23
Biplanar transrectal ultrasound	2.45±0.76	2.45±0.72	0.43±0.22	0.34±0.26
*t*-value	0.920	1.333	0.704	0.657
*P*-value	0.363	0.190	0.485	0.515

## Data Availability

The data and supportive information are available within the article.

## LIST OF ABBREVIATIONS

SUI: Stress urinary incontinence
TPUS: Transperineal pelvic floor ultrasound
BMI: Body mass index
MRI: Magnetic Resonance Imaging

## STANDARDS OF REPORTING

STROBE guidelines were followed.
